# Ethical issues related to human papillomavirus vaccination programs: an example from Bangladesh

**DOI:** 10.1186/s12910-018-0287-0

**Published:** 2018-06-15

**Authors:** Marium Salwa, Tarek Abdullah Al-Munim

**Affiliations:** 1 Department of Public Health and Informatics, Bangabandhu Sheikh Mujib Medical University, Dhaka, Bangladesh; 2 Independent Consultant, Dhaka, Bangladesh

**Keywords:** Human papilloma virus (HPV) vaccination, Ethical issues, Bangladesh, Adolescent health, Cervical cancer

## Abstract

**Background:**

Human Papilloma Virus (HPV) vaccine was introduced in Bangladesh through the arrangement of a demonstration project in Gazipur district in 2016, targeting grade five female students and non-school going girls (age range 10–12 years). HPV vaccination is expected to be eventually included in the nationwide immunization program if the demonstration project is successful. However, introduction and implementation of such a vaccination program raises various ethical concerns. This review paper illustrates a step by step assessment of the ethical concerns surrounding the HPV vaccination implementation in Bangladesh considering specific elements in administering and conducting the program as well as the intended results. Policy-makers, vaccine implementers, vaccine recipients, and an ethics specialist in Bangladesh were interviewed. Electronic database and websites have also been reviewed for relevant published literature and government statements**.**

**Main body of the abstract:**

This program imparted inadequate knowledge about HPV and cervical cancer to the recipients and participants. There was lack of autonomous and informed choice of the girls and their parents about taking the vaccine. The program did not have any follow-up plan for the adverse effects in the long run. The impact of a female-only strategy in the larger societal context was overlooked. There was lack of awareness among the implementers about safeguarding the ethical issues pertaining to HPV vaccination.

**Conclusion:**

Adolescent health education imparted in the scope of the vaccination program should contain adequate information about HPV, its mode of transmission, risk factors along with the importance of secondary prevention despite primary prevention. Adolescent boys should be given HPV related health education as well. The right of making informed choice should be appreciated and respected. More ethical discussion and debate should be done among the public health professionals of Bangladesh in order to increase awareness about ethical issues related to human health.

## Background

Institutionalized vaccination programs have become part of routine health care services for the Bangladeshi masses. The success of polio and smallpox vaccination programs has been a boon for the population of this country and has greatly increased trust in the system to protect the population from debilitating or deadly diseases. The trust in vaccination as an almost guaranteed protection from the disease for which the vaccine is administered is ironclad. Against this backdrop, in 2016, the human papillomavirus (HPV) vaccine was introduced in Bangladesh through the implementation of a demonstration project in Gazipur district, which is located close to the capital city of Dhaka. This demonstration project is governed by the Ministry of Health and Family Welfare of Bangladesh through the Expanded Program on Immunization (EPI), with financial support from the Global Alliance for Vaccines and Immunization (GAVI, the Vaccine Alliance). Eventually HPV vaccination is expected to be included in the nationwide immunization program if the demonstration project is deemed successful.

HPV infection is now a global concern. According to the HPV Information Centre, more than a half-million cancers—including cervical, vulvar, anal, penile, and oropharyngeal cancer—and over 250,000 deaths are attributed annually to HPV infection [1]. Globally, cervical cancer is the fourth leading female cancer and the second most common cancer among women aged 15 to 44 years [1]. However, information about HPV infection in Bangladesh is scarce. According to a study in 2014, the prevalence of HPV infection was 7.7% in Bangladesh [2]. Systematic reviews and meta-analyses have shown the prevalence of HPV in the South Asian region to be lower than in other areas of the world [3, 4]. Despite the comparatively low prevalence of HPV in Bangladesh, the lack of resources for secondary prevention and the high cost of treatment have resulted in about 10,000 cervical cancer deaths every year [5], making it the second most common cancer among women in Bangladesh [6].

HPV is a sexually transmitted viral infection (STI) and is associated with various social stigmas and misconceptions [7, 8]. Also, the HPV vaccination program only targets women, and that, too, at adolescent age. In Bangladesh, vaccinating adolescent girls against an STI is a challenge and raises several ethical issues that need to be assessed. In this article we present a step-by-step assessment of the ethical concerns surrounding implementation of the HPV vaccination program in Bangladesh; we consider specific elements involved in administering and conducting the program, as well as the intended results. We have been inspired to write this article after our experiences with an ongoing documentation study on the current HPV vaccination demonstration project; that said, however, this article is not related to that study. To write this article, we interviewed policy makers, vaccine implementers, vaccine recipients, and an ethics specialist in Bangladesh. Also, electronic database and websites have been searched for relevant published literature and government statements.

## Main text

### About the HPV vaccination demonstration program

The government of Bangladesh has initiated a two-year demonstration program of HPV vaccination in partnership with Gavi, which provides funding for the vaccine, and the United Nations Children’s Fund (UNICEF), which oversees the procurement of the vaccine. Cervarix, the bivalent preparation of the HPV vaccine, is administered in two doses, six months apart, based on the recommendation of the World Health Organization (WHO) [9]. The program involves vaccinating both grade 5 female students and girls who are not attending schools (ages 10–12 years), in five selected areas of Gazipur district. The vaccination program is being conducted under the EPI using its existing resources and is mainly a school-based program. Dates of scheduled vaccinations are announced ahead of time so that all female students can attend. Before administering the vaccine, a health education session on adolescent health and HPV and cervical cancer is conducted among the vaccine candidates. Every girl is then taken separately to a predesignated place for vaccination within the school premises, such as an empty classroom or a secluded corner, to minimize anxiety about vaccination among the other girls. A vaccination card is maintained for every recipient, and the cards are preserved by the school authorities. Those who are absent on the vaccination day can get the vaccine from a nearby school on another date or from the community EPI centre. Furthermore, adolescent girls of this age who are not attending school can get the vaccine from community-based routine, fixed, and outreach vaccination sites. The first round of vaccination was completed in 2016 and was comprised of two doses given six months apart (April and October 2016). The coverage was as high as 94% [10]. National scale-up of the HPV vaccination program is planned for 2019 after the HPV demonstration program is evaluated, assuming it is deemed successful.

### Discussion of ethical issues pertaining to the HPV vaccination demonstration program

#### Ethical issues related to tailored information dissemination about HPV and cervical cancer

Most HPV vaccine recipients, through the health education they received prior to being vaccinated, were made aware about the vaccine and its importance in preventing cervical cancer. However, information about HPV infection, its route of transmission, and the cofactors related to its spread, along with other cervical cancer prevention strategies, were left out of this health education.

HPV is the aetiological cause of cervical cancer. HPV 16 and 18 serotypes, especially, are responsible for about 70% of cervical cancer worldwide [9]. However, infection with HPV does not necessarily result in cervical cancer. There are some established cofactors for progression of cervical HPV infection to cancer in the long run, such as early age of first sexual intercourse, multiple sexual partners, tobacco use, immune suppression such as co-infection with human immunodeficiency virus (HIV), high parity, long-term hormonal contraceptive use, and poor nutritional status [1, 9]. HPV infection can be prevented primarily through vaccination and interventions targeting these modifiable risk factors. Among secondary prevention methods, cervical screening tests show great promise, especially the Papanicolaou test (Pap test) and the HPV determination test offered in resource-rich areas [11], whereas in resource-poor settings the visual inspection with acetic acid (VIA) test or HPV DNA test are established as low-cost and efficient alternatives to cytological testing [12]. In Bangladesh, even though cervical screening programs began in 2004, the coverage rate remains very poor and is limited to opportunistic tests [13]. In this local context, conveying tailored and incomplete information about HPV and cervical cancer to the population raises ethical concerns. Here we present the contexts in which the practice of providing tailored information becomes overt.

First, the fact that sexual contact is the main route of transmission of the vaccine-preventable HPV serotypes is seemingly not openly disclosed to the vaccine recipients, their parents/guardians, or teachers. Not revealing the nature of transmission of the virus against which the girls are being vaccinated raises ethical concerns. There may be scenarios where incomplete information is provided to avoid sociocultural controversies for the greater good. Regarding vaccination, which essentially has a utilitarian benefit, it could be argued that full information might hinder vaccine uptake. Experiences from several low- and middle-income countries (LMICs) have suggested that highlighting the anti-cancer role of the HPV vaccine to the public without mentioning its true role in preventing sexually-transmitted HPV infection is associated with high vaccination coverage [14]. Achieving high vaccination coverage among this adolescent age group is a challenge, as they are older than the usual EPI vaccination target groups and there is no adolescent-friendly healthcare system. So, it could be argued that if providing only partial information results in higher vaccination coverage, this ethical compromise is a small price to pay for the greater good of reaching more girls through the vaccination program.

The alternative to providing framed partial information is providing full information to enable well-informed choices. Some studies suggest there is no significant effect of information framing on HPV vaccine acceptance [11]. However, accurate and complete information is proven to be effective against rumour and misinformation [14]. With regard to STIs, full information may be necessary to avoid marginalizing any specific gender, in social settings that have historically victimized females for many STI issues [15]. Also, due to the lack of public discourse and the sensitivities around making STI-related information readily available, there is little opportunity to command people’s full, undivided attention on multiple occasions; therefore, it is only convenient to provide full information when the opportunity arises, in order to avoid presenting only partial information that can produce a false sense of safety when no such guarantees exist. In fact, revealing the nature of HPV transmission, even though it may raise feelings of stigma and shame, is proven to reduce the stress [16]. Also, the success of the measles rubella (MR) campaign in increasing MR vaccination coverage in Bangladesh demonstrates that good social mobilization in which the recipient is able to learn about the disease in question can result in high vaccine uptake [17].

Second, not providing information about other cervical cancer prevention strategies, particularly cervical screening, poses ethical questions. Vaccination is not a substitute for screening. The vaccine only prevents 70% of cervical cancers, so surveillance via screening must continue [9]. It has been proven that screening for cervical cancer is indispensable [9], even in situations where HPV vaccination coverage is high; yet even so, more weight is being given to HPV vaccination than to creating awareness about the importance of screening and the root causes of HPV. There is a risk that such over-emphasis on HPV vaccination may divert the recipients’ attention from the perceived importance of screening. It is anticipated that some vaccinated women may forego the recommended screening due to this false sense of security, a situation that may paradoxically result in a higher incidence of cervical cancer if less than 70% of the population is screened [18]. It is now recognized from several studies that a comprehensive strategy of combining HPV vaccination at an early age before sexual exposure with subsequent cervical screening three to five times in a woman’s lifetime is more efficient and cost-effective in preventing cervical cancer than vaccination alone [19, 20]. The effectiveness of HPV vaccination is higher where there is a strong screening system. Although Bangladesh has good central co-ordination and some elements of organized screening, its cervical cancer screening program has scored very low coverage, mainly due to lack of social awareness and a weak program strategy [13]. Also, there is no quality assurance structure or clear mandate assigned for supervising and monitoring the screening process [1]. Under these circumstances, the ethically charged contention is raised that over-advertising of vaccination would further diminish the importance of screening in the minds of the population.

Third, there is an ethical dilemma regarding the actual efficacy of the vaccine [21]. Communications around the HPV vaccination program convey the impression that cervical cancer is 100% preventable if the vaccine is taken. Yet the vaccine currently provided under the program only covers two serotypes of HPV [16, 18] out of about 20 possible high-risk cancer-causing serotypes [22]. Also, “more than 80% of lesions with borderline and mildly abnormal cytology and more than 70% with low grade abnormality on histology (cervical intraepithelial neoplasia 1 and 2) are not related to HPV-16 or HPV-18 and so will not be prevented by vaccination” [23]. Interviews with recipient girls suggested they believed this vaccine would prevent cervical cancer, just as the polio vaccine prevents polio. Parallels have been drawn by the recipients between polio and HPV, which may well have been the modus operandi for promoting the program to parents and children, but which is unethical given the clear dissimilarities between the two. Even for the two serotypes for which the vaccination is effective, protection from HPV infection is assumed to be long-lasting [24, 25], but the accurate duration of protection is yet to be evaluated [21]. Some scientists believe booster doses will be required after a couple of years to ensure efficacy of the vaccine [26]. A matter of concern is that the introduction of booster doses is not within the program’s current scope. The program communication also does not disclose adequate information about the durability of protection and the need for probable booster immunization. This is undeniably an ethical concern, as there is a clear lack of accountability that risks the loss of public trust regarding preventive health services, which may fail miserably for many families going forward.

#### Ethical issues related to making autonomous and informed choices

Obtaining consent from adolescents or their parents/guardians before giving vaccination is one of the most challenging tasks in the HPV vaccination program. Adolescence is a distinctive life stage, and hence any health intervention at this age requires special attention. WHO stresses the particular need to obtain informed consent when administering vaccination for this age group [27]. Many HPV vaccination programs around the world, including in Bangladesh, use an implied consent procedure. Implied consent means parents/guardians are informed about imminent vaccination through social mobilization and communication [28], and thus if they send their child/ward to the vaccination session, it is assumed they want them to receive the vaccine, and vice versa. Yet whether consent is implied or written, it should be well informed [28].

In Bangladesh, there is no written guideline or procedure on ensuring the obtaining of parental/guardian consent or the assent of the adolescents for vaccination. In usual practice, it is expected that adolescents are accompanied by their parents/guardians when undergoing any health intervention at health service centres. However, there is no recommendation regarding non-accompanied adolescents receiving vaccines. This raises ethical questions around whether adolescent vaccine recipients can provide their own consent and how vaccination can be ensured while balancing parental/guardian consent with the assent of recipients. At school, adolescent girls are vaccinated without the presence of their parents/guardians based on implied parental/guardian consent. The assent of the girls, which is considered important for vaccination according to WHO [28], is not covered under the procedures.

In the HPV vaccination demonstration program of Bangladesh, the social mobilization carried out in the vaccination areas has been very poor, both in quality and quantity. Many parents/guardians are unaware of the vaccine their daughters/wards have received at school. One positive feature of the program is that the girls are taught about the HPV vaccine and cervical cancer through a health education session before receiving the vaccine. Although partial, this education helps girls to know something about the vaccine they are going to receive. However, given the regimented way in which the vaccination is administered in schools, all selected girls feel obliged to accept it, either due to peer pressure or to please school teachers. They have no opportunity to take an informed and independent decision regarding the matter. This creates an ethical dilemma, given that no choice, in its truest sense, is available to the recipients or their parents/guardians when deciding whether to take the vaccine. For adolescent girls not attending school, coming to the EPI centres for vaccination is considered as giving implied consent. In reality, however, they are lured into taking the vaccine by hearing it referred to as the ‘cervical cancer vaccine’ during community mobilization, and complete information about the vaccine and its limitations is not disclosed.

Immunization of all eligible girls to create herd immunity may be rationalized by the theory of utilitarianism, which considers an action’s rightness or wrongness based on its consequences [29]. However, the low indication that this is indeed the most effective means of preventing cervical cancer in the context of Bangladesh makes this lack of autonomous choice ethically dubious. It is particularly important that informed consent be obtained from the parents/guardians for vaccination of adolescents [11]. It is their right, after getting adequate information about the vaccination and its consequences, to either refuse or allow their daughters/wards to be vaccinated. Studies suggest that involving parents/guardians in the discussion at the time of vaccination enhances communication about cervical cancer prevention and the particular role of the HPV vaccine [30]. However, this important issue seems to be ignored in the current vaccination program.

Furthermore, an HPV-vaccination card is maintained for every vaccine recipient, but these are not given to the girls; rather, they are deposited with the relevant school authorities until the two doses are completed. This raises another dimension of ethical concern. Although the reason provided, at the implementer level, for not giving the vaccination cards to the girls is to protect the cards from being lost, it is still a matter of concern that there is no written documentation for the parents/guardians to know about the vaccine administered to their daughters/wards at school. This puts parents/guardians in a blind situation, making them solely dependent on the school teachers’ judgment about the well-being of their daughters/wards and depriving them of being able to make their own decisions.

Studies suggest that effective and thorough social mobilization with accurate and complete knowledge about HPV ultimately results in successful vaccination programs in the long run [14]. Also, vaccination programs that are accessible, well-communicated, and supported by law and that allow parents/guardians to make informed decisions are proven to counter misinformation about vaccination. This suggests that well-organized social mobilization with enriched communication materials should be executed in effective ways before starting any vaccination campaign. Also, the health education session needs to be more informative and understandable to the girls so that they are empowered to take informed decisions about other preventive measures, besides vaccination, for cervical cancer and HPV infections.

#### Ethical concerns related to follow-up of the vaccinated cohort

Interviews with policy-makers revealed that the HPV vaccine and all the EPI vaccines are used in Bangladesh with WHO approval but without any country-specific trial or licensure by any local drug administration authority. Hence post-marketing vaccine surveillance is poor. This is a concern because, whereas most EPI vaccines’ adverse effects are immediately identified, there may long-term adverse effects associated with cases of HPV vaccinations. Consequently, not performing due diligence in the context of Bangladesh raises ethical questions, given the risks associated with HPV vaccinations, which for the most part is still unknown.

Any adverse effect following immunization (AEFI) with HPV vaccine is recorded using an existing EPI form. The EPI form is adequate for EPI listed vaccines, as it captures immediate and well-established adverse effects following immunization. On the other hand, HPV vaccinations may not necessarily result in short-term adverse effects, but in long-term ones yet to be evaluated, which cannot be recorded on the current EPI form. The pharmaceutical manufacturers have reported “local pain” as the most common side-effect of the HPV vaccine [25, 31]. However, more serious AEFIs, although rare, have been reported in other studies [32]. Thus long-term follow up of HPV vaccine recipients is essential and recommended by the Centers for Disease Control and Prevention [33]. Depriving the vaccinated girls of more organized long-term follow-up, and hence not ensuring immediate identification and treatment of any vaccine-related adverse effects, poses a major ethical concern.

There is still a lack of data on how long the vaccine will give immunity or on the need for any booster dose [21, 26]. As such, the vaccinated cohort would need to be followed for several decades [34] to know more precisely the efficacy of the vaccine, any unintended side effects, the duration of protection, and the necessity for booster doses, as well as to monitor HPV serotypes in cervical intraepithelial lesions. Furthermore, since there is no country-specific data from Bangladesh, reliable record-keeping is needed on the part of both the HPV administration and the vaccine recipient. On the administration’s side, this requires significant technical and financial readiness to ensure proper and sound recordkeeping for every vaccine recipient. This is not easy given the existing infrastructure. For the recipient, except for the financially solvent, such recordkeeping entails the challenge of preserving hard-copy documents over the long haul, with high risks of documents being damaged or lost; recovering such documents will also require technical support from the administration, adding to the system complexity. Putting in place the above technical and system requirements at the outset would have made it easier to use the data for study and analysis; this failure is another drawback of the existing reporting system.

#### Ethical impact of a female-only strategy on society

The HPV vaccination program targets only the young female population, whereas the male population, who are in effect a major source of transmission, are left out of the program. Globally the HPV vaccination program targets women to minimize the overall cost of the program [35], although there is significant evidence to justify male HPV vaccination [24]. Vaccinating men and boys against HPV could play a role in increasing herd immunity and decreasing the overall incidence of cervical cancer [36]. Even though male inclusion in the HPV vaccination program has been shown to increase the overall cost, the benefit is also proven to be high compared to female-only vaccination [36]. However, including males in the current vaccination program along with females is not the topic of our discussion; rather, our aim is to analyze this from an ethical standpoint and in the perspective of Bangladesh.

The gender split of the Bangladeshi population is approximately 50–50 [37]. Even though equal rights for women have progressed and women’s inclusion in the workforce has grown significantly over the years [38], there remain significant economic and cultural barriers that women have yet to overcome. That said, there are signs that this rise in women’s empowerment has not lessened the violence against women [39]. Also, their increased representation in the government and non-government sectors is not associated with an increased presence of women in the decision-making process [40]. Females are already victimized for many medical conditions that are unexplained or result from their male partner’s behaviour. Any additional intervention that may aggravate victimization and stigma against women would, in this context, be unethical and counterproductive. Furthermore, in Bangladesh, especially in the rural context, vaccinating only females may lead people to dismiss the disease as a women-related concern. The corollary of this view would be that any woman in whom the disease is identified would be considered at fault. When a grade 5 boy sees his female classmates taken to another room for special health education and to be vaccinated with HPV vaccine, it would not be surprising that the boy would draw wrong conclusions regarding HPV and cervical cancer. From a very early age, this boy will learn to identify HPV, and thus cervical cancer, as a merely women-related issue, which may give him a false sense of security. In reality, males are the important link in the epidemiological chain of HPV transmission and can suffer from HPV infection as well.

This female-only approach may also increase sexual irresponsibility in the male partner, especially given that, in Bangladesh, females generally have less of a say regarding choices in sex. Women’s significant progress in matters such as education and financial independence has already produced examples, in Bangladesh, of insecurity on the part of their male partners that has, at times, led to deadly violence. The asymmetrical way in which vaccination is being conducted globally, targeting only females for purported cost–efficiency reasons, will gradually build a misconception that HPV infection is a female-only matter, which may have negative consequences on the larger social psyche that already marginalizes women.

## Conclusion

Vaccination is a core delivery of public health services. In Bangladesh, the immunization program still holds the public’s trust and is regarded as the most successful public health program in the country. Hence, to preserve ethical standards, adding any new vaccine to the existing program requires thorough investigation of its compatibility, necessity, and fit-for-purpose. To alleviate the concerns raised in this paper, we offer the following recommendations. Adolescent health education imparted in the scope of vaccination should contain adequate information about HPV infection and cervical cancer, as well as other measures to prevent cervical cancer, such as safe personal practices, including use of the barrier method, delayed sexual debut, smoking cessation, regular cervical screening, etc. Any misleading or partial information should be identified and prohibited. The procedure for obtaining parental/guardian consent while ensuring adolescents’ autonomy and assent to vaccination should be established and well-circulated among health service providers. The vaccinated cohort should be kept under long-term follow-up. Adolescent boys should also be given health education regarding HPV-related infection. More ethical discussion and debate are needed among public health professionals in Bangladesh to increase awareness about ethical issues related to human health.

## Abbreviations

AEFI: Adverse Effect Following Immunization
EPI: Expanded Program on Immunization
GAVI: Global Alliance for Vaccines and Immunization
HIV: Human immunodeficiency virus
HPV: Human papillomavirus
LMICs: Low- and middle-income countries
MR: Measles rubella
STI: Sexually transmitted infection
UNICEF: United Nations Children’s Fund
VIA: Visual inspection with acetic acid
WHO: World Health Organization
